# Practical Stability in terms of Two Measures for Set Differential Equations on Time Scales

**DOI:** 10.1155/2014/241034

**Published:** 2014-01-20

**Authors:** Peiguang Wang, Weiwei Sun

**Affiliations:** ^1^College of Electronic and Information Engineering, Hebei University, Baoding 071002, China; ^2^College of Mathematics and Computer Science, Hebei University, Baoding 071002, China

## Abstract

We present a new comparison principle by introducing a notion of upper quasi-monotone nondecreasing and obtain the practical stability criteria for set valued differential equations in terms of two measures on time scales by using the vector Lyapunov function together with the new comparison principle.

## 1. Introduction

Stability theory in the sense of Lyapunov is now well known. Its basic theory and applications can be found in the monographs of Lasalle and Lefschetz [1] and Rouche et al. [2]. To unify a variety of stability concepts and to offer a general framework for the investigation of the stability theory, the notion of stability in terms of two measures has been proved to be very powerful (see the monograph [3] and the papers [4–6]).

The practical stability is a very important problem in the field of application, which deals with the question of whether the system state evolves within certain subsets of the state-space. It is very useful in estimating the worst-case transient and steady-state responses and in verifying pointwise in time constraints imposed on the state trajectories. Thus practical stability is concerned with quantitative analysis as opposed to Lyapunov analysis which is qualitative in nature. There are some relative results for practical stability of various dynamic systems. We can refer to the monograph of Lakshmikantham et al. [7] and the papers of Zhang and Sun [8], Wang and Liu [9], Wang et al. [10], Sun et al. [11], and Hristova and Georgieva [12] and the references cited therein.

Recently, the study of set differential equations in a semilinear metric space has gained much attention due to its applicability to multivalued differential inclusions and fuzzy differential equations and its inclusion of ordinary differential systems as a special case [13], and some basic results of interest are obtained in [14–21]. However, we notice that there are very few results for set valued differential equation on time scales. For example, Girton [22] gave the results of the existence and uniqueness of the solution of an initial value problem that involve set valued differential equations on time scales. Ahmad and Sivasundaram [23] and Hong [24, 25] discussed some basic problems of set valued differential equation on time scales and obtained some stability criteria, respectively. In this paper, we present a new comparison principle by introducing a notion of upper quasi-monotone nondecreasing and obtain the practical stability criteria for set valued differential equations in terms of two measures on time scales by using the vector Lyapunov function together with the new comparison principle. Consequently, this paper is organized as follows. In Section 2, we introduce the concepts of the time scales and the set valued differential equations, the propositions of the set valued differential equations on time scales. In Section 3, the relatively new comparison principle and conditions of stability are given.

## 2. Preliminaries

Let *𝕋* be a time scale with *t*_0_ ≥ 0 as minimal element and no maximal element. Firstly, we give some relative definitions, which can be found in [1–3].

**Definition 1**

The mappings *σ*, *ρ*: *𝕋* → *𝕋* defined as

(1) $$\begin{array}{c} \sigma \left(t\right)=\inf\left(s\in 𝕋;s>t\right),\;\;\rho \left(t\right)=\sup\left(s\in 𝕋;s<t\right) \end{array}$$

are called jump operators.

**Definition 2**

A nonmaximal element *t* ∈ *𝕋* is said to be right-scattered (rs) if *σ*(*t*) > *t* and right-dense (rd) if *σ*(*t*) = *t*. A nonminimal element *t* ∈ *𝕋* is called left-scattered (ls) if *ρ*(*t*) < *t* and left-dense (ld) if *ρ*(*t*) = *t*.

**Definition 3**

Let

(2) $$\begin{array}{c} {𝕋}^{k}=\{\begin{array}{cc} 𝕋\setminus \left(\rho \left(\sup 𝕋\right),\sup 𝕋\right) & \text{if}\;\;\sup 𝕋<\infty ,\\ 𝕋 & \text{if}\;\;\sup 𝕋=\infty . \end{array} \end{array}$$

**Definition 4**

The mapping *g* is called regulated, if in each left-dense *t* ∈ *𝕋* the left sided and in each right-dense *t* ∈ *𝕋* the right sided limit exist.

**Definition 5**

The mapping *g* : *𝕋* → ℝ^*n*^ is called rd continuous if

- it is continuous at each right-dense *t* ∈ *𝕋*;
- at each left-dense point the left-sided limit *g*(*t* ^−^) exists.

Let *C*_*rd*⁡_[*𝕋*, ℝ^*n*^] denote the set of rd-continuous mappings from *𝕋* to ℝ^*n*^.

**Definition 6**

The mapping *f* : *𝕋* × ℝ^*n*^ → ℝ^*n*^ is said to be right-dense (rd) continuous and is denoted by *f* ∈ *C*_*rd*⁡_[*𝕋* × ℝ^*n*^, ℝ^*n*^] if

- it is continuous at each (*t*, *x*) with right-dense or maximal *t*;
- the limits *f*(*t* ^−^, *x*) = lim⁡_(*s*,*y*)→(*t*^−^,*x*)_ *f*(*s*, *y*) and lim⁡_*y*→*x*_ *f*(*t*, *y*) exist at each (*t*, *x*) with left-dense *t*.

Let *K*(ℝ^*n*^) denote the collection of all nonempty, compact, and convex subsets of ℝ^*n*^. Define the Hausdorff metric

(3) $$\begin{array}{c} D\left[A,B\right]=\max\left[\underset{x\in B}{\sup}\;d\left(x,A\right),\underset{y\in A}{\sup}\;d\left(y,B\right)\right], \end{array}$$

where *d*[*x*, *A*] = inf⁡[*d*(*x*, *y*) : *y* ∈ *A*], *A*, *B* are bounded sets in ℝ^*n*^. We note that *K*(ℝ^*n*^) with this metric is a complete metric space.

It is known that if the space *K*(ℝ^*n*^) is equipped with the natural algebraic operations of addition and nonnegative scalar multiplication, then *K*(ℝ^*n*^) becomes a semilinear metric space which can be embedded as a complete cone into a corresponding Banach space.

The Hausdorff metric (3) satisfies the following properties:

(4) $$\begin{array}{c} D\left[A+C,B+C\right]=D\left[A,B\right],\;\;D\left[A,B\right]=D\left[B,A\right],\\ D\left[\lambda A,\lambda B\right]=\lambda D\left[A,B\right],\\ D\left[A,B\right]\leq D\left[A,C\right]+D\left[C,B\right], \end{array}$$

for all *A*, *B*, *C* ∈ *K*(ℝ^*n*^) and *λ* ∈ ℝ_+_.

**Definition 7**

Let *A*, *B* ∈ *K*(ℝ^*n*^). The set *C* ∈ *K*(ℝ^*n*^) satisfying *A* = *B* + *C* is known as the Hukuhara difference of the sets *A* and *B* and is denoted by the symbol *A* − *B*.

**Definition 8**

The forward derivative of *F*: *𝕋* → *K*(ℝ^*n*^), denoted by Δ*F*, is defined by

(5) $$\begin{array}{c} \Delta F\left(t\right)=\frac{F\left(\sigma \left(t\right)\right)-F\left(t\right)}{\sigma \left(t\right)-t},\;\;\text{if}\;t\;\text{is}\;\text{right-scattered},\\ \Delta F\left(t\right)=\underset{s\to t^{+}}{\lim}\frac{F\left(s\right)-F\left(t\right)}{s-t},\;\text{if}\;t\;\text{is}\;\text{right-dense}. \end{array}$$

Analogously, the backward derivative ∇*F* is defined as

(6) $$\begin{array}{c} \nabla F\left(t\right)=\frac{F\left(t\right)-F\left(\rho \left(t\right)\right)}{t-\rho \left(t\right)},\;\text{if}\;t\;\text{is}\;\text{left-scattered},\\ \nabla F\left(t\right)=\underset{s\to t^{-}}{\lim}\frac{F\left(t\right)-F\left(s\right)}{t-s},\;\text{if}\;t\;\text{is}\;\text{left-dense}. \end{array}$$

**Remark 9**

The usual scalar subtraction occurs in the scalar coefficient at the beginning of the above expressions and the Hukuhara set difference occurs inside the brackets. Clearly, the existence of Hukuhara difference ensures the existence of the derivative in Definition 8; in the forthcoming analysis, we will be considering the forward derivative as the result for backward derivative followed immediately with suitable changes. The backward derivative has the following properties:

- let *F*, *G* : *𝕋* → *K*(ℝ^*n*^) be differentiable at *t*; then Δ(*F* + *G*)(*t*) = Δ*F*(*t*) + Δ*G*(*t*);
- Δ(*αF*)(*t*) = *α*(Δ*F*(*t*)), *α* ∈ ℝ_+_.

**Remark 10**

If the sets in *K*(ℝ^*n*^) are singletons only, then there is only one selector possible, namely, *F* itself. In this case, the integral reduces to the generalized integral from time scales into (ℝ^*n*^). The assumption *n* = 1 generalizes into the integral for time scales. When *𝕋* is everywhere right-dense, then we have Δ*t* = *dt*, which results in the conventional formulation for integration of set valued functions. Moreover, for *a*, *b*, *c* ∈ *𝕋*, we have

- ∫_*a*_^*c*^ *F*(*t*)Δ*t* = ∫_*a*_^*b*^ *F*(*t*)Δ*t* + ∫_*b*_^*c*^ *F*(*t*)Δ*t*;
- ∫_*a*_^*b*^ *F*(*t*)Δ*t* is closed and convex but need not be necessarily compact.

**Definition 11**

Let *F*, *G*: *𝕋* → *K*(ℝ^*n*^). Then the Hausdorff distance between *F* and *G*, *D*[*F*(·), *G*(·)]: *𝕋* → ℝ_+_, is Δ-integrable and

(7) $$\begin{array}{c} D\left[\overset{t}{\underset{t_{0}}{\int }}F\left(s\right)\Delta s,\overset{t}{\underset{t_{0}}{\int }}G\left(s\right)\Delta s\right]\leq \overset{t}{\underset{t_{0}}{\int }}D\left[F\left(s\right),G\left(s\right)\right]\Delta s. \end{array}$$

**Definition 12**

Assume *F*: *𝕋* → *K*(ℝ^*n*^) is set valued function and *t* ∈ *𝕋* ^*k*^. Let Δ_*H*_ *F*(*t*) be an element of *K*(ℝ^*n*^) (provided it exists) with the property that given any *ϵ* > 0, there exists a neighborhood *U*_*t*_ of *t* (i.e., *U*_*t*_ = (*t* − *δ*, *t* + *δ*)⋂*𝕋* for some *δ* > 0) such that

(8) D[F(t+h)−f(σ(t)),ΔHF(t)(h−μ(t))]  ≤ϵ(h−μ(t)),D[f(σ(t))−F(t−h),ΔHF(t)(μ(t)+h)]  ≤ϵ(μ(t)+h)

for all (*t* − *h*, *t* + *h*) ∈ *U*_*t*_ with 0 ≤ *h* < *δ*, where *μ*(*t*) = *σ*(*t*) − *t*. We call Δ_*H*_ *F*(*t*) the Δ_*H*_-derivative of *F* at *t*. We say that *F* is Δ_*H*_-differentiable at *t* if its Δ_*H*_-derivative exists at *t*. Moreover, we say *F* is Δ_*H*_-differentiable on *𝕋* ^*k*^ if its Δ_*H*_-derivative exists at each *t* ∈ *𝕋* ^*k*^. The multivalued function Δ_*H*_ *F*(*t*): *𝕋* ^*k*^ → *K*(ℝ^*n*^) is then called the Δ_*H*_-derivative of *F* on *𝕋* ^*k*^.

**Proposition 13**

*Let* *F*: *𝕋* → *K*(ℝ^*n*^), Δ_*H*_ *F*(*t*) ∈ *K*(ℝ^*n*^)  *(provided it exists). Then some easy and useful relationships concerning the *Δ_*H*_ *-derivative hold.*

(i) *If the*  Δ_*H*_-*derivative of* *F* *at* *t* *exists, then it is unique. Hence, the*  Δ_*H*_ *-derivative is well defined.*
(ii) *Assume* *F:* *𝕋* → *K*(ℝ^*n*^) *is a multivalued function and let* *t* ∈ *𝕋* ^*k*^ *. Then one has the following.*
  (1) *If* *F* *is*  Δ_*H*_ *-differentiable at* *t, then* *F* *is continuous at* *t*.
  (2) *If* *F* *is continuous at* *t* *and* *t* *is right-scattered, then* *F* *is*  Δ_*H*_ *-differentiable at* *t* *with*
    (9) $$\begin{array}{c} \Delta_{H}F\left(t\right)=\frac{F\left(\sigma \left(t\right)\right)-F\left(t\right)}{\mu \left(t\right)}. \end{array}$$
  (3) *If* *t* *is right-dense, then* *F* *is*  Δ_*H*_ *-differentiable at* *t* *if the limits*
    (10) $$\begin{array}{c} \underset{h\to 0^{+}}{\lim}\frac{F\left(t+h\right)-F\left(t\right)}{h},\;\;\underset{h\to 0^{+}}{\lim}\frac{F\left(t\right)-F\left(t-h\right)}{h} \end{array}$$
   
*exist and satisfy the equations *
    (11) $$\begin{array}{c} \underset{h\to 0^{+}}{\lim}\frac{F\left(t+h\right)-F\left(t\right)}{h}=\underset{h\to 0^{+}}{\lim}\frac{F\left(t\right)-F\left(t-h\right)}{h}=\Delta_{H}F\left(t\right). \end{array}$$
  (4) *If* *F* *is differentiable at* *t, then*
    (12) $$\begin{array}{c} F\left(\sigma \left(t\right)\right)=F\left(t\right)+\mu \left(t\right)\Delta_{H}F\left(t\right). \end{array}$$

**Remark 14**

Let *F*: *𝕋* → *K*(ℝ^*n*^), Δ_*H*_ *F*(*t*) ∈ *K*(ℝ^*n*^) (provided it exists). We consider the two cases *𝕋* = ℝ and *𝕋* = *ℤ*, where *ℤ* stands for the set consisting of all integers.

(1) If *𝕋* = ℝ, then *F*: ℝ → *K*(ℝ^*n*^) is Δ_*H*_-differentiable at *t* ∈ ℝ if and only if
  (13) $$\begin{array}{c} D_{H}F\left(t\right)=\underset{h\to 0^{+}}{\lim}\frac{F\left(t+h\right)-F\left(t\right)}{h}=\underset{h\to 0^{+}}{\lim}\frac{F\left(t\right)-F\left(t-h\right)}{h} \end{array}$$

exists, that is, if and only if *F* is differentiable in the Hukuhara sense at *t*. In this case, we have

(14) $$\begin{array}{c} D_{H}F\left(t\right)=\Delta_{H}F\left(t\right). \end{array}$$

(2) If *𝕋* = *ℤ*, then *F*: *ℤ* → *K*(ℝ^*n*^) is Δ_*H*_-differentiable at *t* ∈ *ℤ* with
  (15) $$\begin{array}{c} \Delta_{H}F\left(t\right)=\frac{F\left(\sigma \left(t\right)\right)-F\left(t\right)}{\mu \left(t\right)}=F\left(t+1\right)-F\left(t\right)=\Delta F\left(t\right), \end{array}$$

where Δ is the usual forward multivalued difference operator.

## 3. Main Results

Consider the space

(16) K(ℝn)N=K(ℝn)×K(ℝn)×K(ℝn),…,K(ℝn),N  times,  N∈Z,

together with either of the following metrics on the space *K*(ℝ^*n*^)^*N*^:

(17) $$\begin{array}{c} \tilde{D}\left[X,Y\right]=\left(D\left[X_{1},Y_{1}\right],D\left[X_{2},Y_{2}\right],\ldots ,D\left[X_{N},Y_{N}\right]\right), \end{array}$$

where *X*, *Y* ∈ *K*_*c*_(*R* ^*n*^)^*N*^, *X*_*i*_, *Y*_*i*_ ∈ *K*_*c*_(*R* ^*n*^), *i* = 1,2,…, *N*.

Consider the initial value problem

(18) $$\begin{array}{c} \Delta_{H}X=F\left(t,X\right),\;X\left(t_{0}\right)=X_{0}, \end{array}$$

where *F* ∈ *C*_*rd*⁡_[*𝕋* × *K*(ℝ^*n*^)^*N*^, *K*(ℝ^*n*^)^*N*^], *X* ∈ *K*(ℝ^*n*^)^*N*^, *X* = (*X*_1_, *X*_2_,…, *X*_*N*_) such that for each *i*, *i* = 1,2,…, *N*, *X*_*i*_ ∈ *K*(ℝ^*n*^).

First of all, we define the following classes of function:

(19) 𝒦={α:ℝ+⟶ℝ+,  α  is  strictly  increasing  and  α(0)=0},𝒞𝒦={ϕ:𝕋×ℝ+⟶ℝ+,ϕ(t,s)∈𝒦  for  each  t},Γ={h:𝕋×K(ℝn)N⟶ℝ+,inf⁡X∈K(ℝn)Nh(t,X)=0},Σ={Q:ℝ+⟶ℝ+,Q(0)=0  and  Q  is  increasing},S(h,γ)={(t,X)∈𝕋×K(ℝn)N,  h∈Γ  and  h(t,X)<γ,γ>0}.

In order to discuss the stability of the solution of set valued differential systems (18), we state some notions and definitions.

On the vector Lyapunov function on time scales, we defined the Dini derivative of the function *V* ∈ *C*_*rd*⁡_[*𝕋* × *K*(ℝ^*n*^)^*N*^, ℝ^*N*^] along with the solutions of (18) by

(20) DΔ−V(t,X)=liminf⁡μ(t)→0−V(t,X)−V(t−μ(t),X(t)−μ(t)F(t,X))μ(t),DΔ+V(t,X)=limsup⁡μ(t)→0+V(t+μ(t),X(t)+μ(t)F(t,X))−V(t,X)μ(t).

**Definition 15**

A function *G*: *𝕋* × ℝ_+_^*N*^ → ℝ^*N*^  (*N* ≥ 1) is said to be upper quasi-monotone nondecreasing in *U*, if *U*, *W* ∈ ℝ_+_^*N*^ and ||*U*||_*M*_ ≤ ||*W*||_*M*_ imply ||*G*(*t*,*U*)||_*M*_ ≤ ||*G*(*t*,*W*)||_*M*_, where ||·||_*M*_ = max⁡_1≤*i*≤*N*_ *U*_*i*_.

In the following, we will prove the comparison result in terms of vector Lyapunov functions relative to the set differential system on time scales.

**Theorem 16**

Assume that

(*H*_1_) *V* ∈ *C*_*rd*⁡_[*𝕋* × *K*(ℝ^*n*^)^*N*^, ℝ^*N*^], *V* is locally Lipschitzian in *X*; that is, for *X*, *Y* ∈ *K*(ℝ^*n*^)^*N*^, one has $\left|V\left(t,X\right)-V\left(t,Y\right)\right|\leq A\tilde{D}[X,Y]$, where *A* is an *N* × *N* matrix of nonnegative elements, |*V*(*t*, *X*) − *V*(*t*, *Y*)| = (|*V*_1_(*t*, *X*) − *V*_1_(*t*, *Y*)|, |*V*_2_(*t*, *X*) − *V*_2_(*t*, *Y*)|,…, |*V*_*N*_(*t*, *X*) − *V*_*N*_(*t*, *Y*)|). Here, by |*V*(*t*, *X*)| one means the vector (|*V*_1_(*t*, *X*)|, |*V*_2_(*t*, *X*)|,…, |*V*_*N*_(*t*, *X*)|), where *V*_*i*_ are the components of *V*, *i* = 1,2,…, *N*;
(*H*_2_) *V* satisfies
  (21) $$\begin{array}{c} D_{\Delta }^{+}V\left(t,X\right)\leq G\left(t,V\left(t,X\right)\right), \end{array}$$

where *G* ∈ *C*_*rd*⁡_[*𝕋* × ℝ^*N*^, ℝ^*N*^] is upper quasi-monotone nondecreasing in *U* for each *t* ∈ *𝕋*;

(*H*_3_) *U* ∈ ℝ^*N*^, *U*(*t*) = *U*(*t*, *t*_0_, *U*_0_) is the solution of
  (22) $$\begin{array}{c} U^{\Delta }=G\left(t,U\right),\;\;U\left(t_{0}\right)=U_{0}. \end{array}$$

Then for any solution *X*(*t*) of (18), one has

(23) $$\begin{array}{c} {||V\left(t,X\right)||}_{M}\leq {||U\left(t\right)||}_{M},\;t\in 𝕋, \end{array}$$

provided ||*V*(*t*_0_,*X*_0_)||_*M*_ ≤ ||*U*_0_||_*M*_.

**Proof**

Let *U*(*t*) be the solution of (22) for *t* ≥ *t*_0_, *t* ∈ *𝕋*. Set *m*(*t*) = *V*(*t*, *X*(*t*)) as an application of the properties of Hausdorff metric; we obtain the estimation

(24) m(σ(t))−m(t) =V(σ(t),X(σ(t)))−V(t,X(t)) =V(σ(t),X(σ(t)))  −V(σ(t),X(t)+(σ(t)−t)F(t,X(t)))  +V(σ(t),X(t)+(σ(t)−t)F(t,X(t)))  −V(t,X(t)) ≤AD~[X(σ(t)),X(t)+(σ(t)−t)F(t,X(t))]  +V(σ(t),X(t)+(σ(t)−t)F(t,X(t)))  −V(t,X(t)).

Letting *X*(*σ*(*t*)) = *X*(*t*) + *Y*(*t*), where *Y*(*t*) is the Hukuhara difference of *X*(*σ*(*t*)) and *X*(*t*) for small *h* > 0 and is assumed to exist, hence,

(25) m(σ(t))−m(t) ≤AD~[X(t)+Y(t),X(t)+(σ(t)−t)F(t,X(t))]  +V(σ(t),X(t)+(σ(t)−t)F(t,X(t)))  −V(t,X(t)) =AD~[Y(t),(σ(t)−t)F(t,X(t))]  +V(σ(t),X(t)+(σ(t)−t)F(t,X(t)))  −V(t,X(t)) =AD~[X(σ(t))−X(t),(σ(t)−t)F(t,X(t))]  +V(σ(t),X(t)+(σ(t)−t)F(t,X(t)))  −V(t,X(t)).

Consequently, we find that

(26) DΔ+m(t)=limsup⁡σ(t)−t→0+m(σ(t))−m(t)σ(t)−t=limsup⁡σ(t)−t→0+1σ(t)−tAD~[X(σ(t))−X(t),           (σ(t)−t)F(t,X(t))] +V(σ(t),X(t)+(σ(t)−t)F(t,X(t)))−V(t,X(t))σ(t)−t≤AD~[X(σ(t))−X(t)σ(t)−t,F(t,X(t))]+DΔ+V(t,X)=AD~[ΔHX(t),F(t,X(t))]+DΔ+V(t,X)=DΔ+V(t,X),

where *D*_Δ_^+^ *m*(*t*) is the right-derivative of *m*(*t*). Hence, we have

(27) $$\begin{array}{c} D_{\Delta }^{+}m\left(t\right)\leq G\left(t,m\left(t\right)\right). \end{array}$$

It is said that

(28) $$\begin{array}{c} {||D_{\Delta }^{+}m\left(t\right)||}_{M}\leq {||G\left(t,m\left(t\right)\right)||}_{M}. \end{array}$$

Because ||*V*(*t*_0_,*X*_0_)||_*M*_ ≤ ||*U*_0_||_*M*_, we can obtain that

(29) $$\begin{array}{c} {||V\left(t,X\right)||}_{M}\leq {||U\left(t\right)||}_{M},\;t\in 𝕋. \end{array}$$

The proof is complete.

Now, we list some definitions about stability which will be used in the following discussion.

**Definition 17**

Let *h*_0_, *h* ∈ Γ, *t* ∈ *𝕋*. Then one says that

(i) *h*_0_ is finer than *h* if there exists a *ρ* > 0 and a function *ϕ* ∈ *𝒞𝒦* such that
  (30) $$\begin{array}{c} h_{0}\left(t,x\right)<\rho \;\text{implies}\;h\left(t,x\right)\leq \phi \left(t,h_{0}\left(t,x\right)\right); \end{array}$$
(ii) *h*_0_ is uniformly finer than *h* if in (i) *ϕ* is independent of *t*.

**Definition 18**

Let *λ*, *A*, *B* be positive constants (*λ* < *A*, *B* < *A*). The system (18) is said to be

- practically stable if for any 0 < *λ* < *A*, the condition ||*X*_0_||_*M*_ < *λ* implies ||*X*(*t*)||_*M*_ < *A*, *t* ≥ *t*_0_ for *t*_0_, *t* ∈ *𝕋*, where *X*(*t*) = *X*(*t*, *t*_0_, *X*_0_) is any solution of (18);
- practically quasi-stable if for any *λ*, *B*, *T* > 0 and some *t*_0_ ∈ *𝕋* with *t*_0_ + *T* ∈ *𝕋*, the condition ||*X*_0_||_*M*_ < *λ* implies ||*X*(*t*)||_*M*_ < *B*, *t* ≥ *t*_0_ + *T*, *t* ∈ *𝕋*;
- strongly practically stable if (i) and (ii) hold simultaneously;
- practically asymptotically stable if (i) holds and for any *ϵ* > 0 there exists *T*_0_ > 0 such that *t*_0_ + *T*_0_ ∈ *𝕋* and ||*X*_0_|| < *λ* implies ||*X*(*t*)|| < *ϵ*, *t* ≥ *t*_0_ + *T*_0_.

**Definition 19**

Let *λ*, *A*, *B* be positive constants (*λ* < *A*, *B* < *A*), *h*, *h*_0_ ∈ Γ. The system (18) is said to be

(PS1) (*h*_0_, *h*)-practically stable if for any 0 < *λ* < *A*, the condition *h*_0_(*t*_0_, *X*_0_) < *λ* implies *h*(*t*, *X*(*t*)) < *A*, *t* ≥ *t*_0_, for some *t*_0_, *t* ∈ *𝕋*, where *X*(*t*) = *X*(*t*, *t*_0_, *X*_0_) is any solution of (18);
(PS2) (*h*_0_, *h*)-practically quasi-stable if for any *λ*, *B*, *T* > 0 and some *t*_0_ ∈ *𝕋* with *t*_0_ + *T* ∈ *𝕋*, the condition *h*_0_(*t*_0_, *X*_0_) < *λ* implies *h*(*t*, *X*(*t*)) < *B*, *t* ≥ *t*_0_ + *T*, *t* ∈ *𝕋*;
(PS3) (*h*_0_, *h*)-strongly practically stable if (PS1) and (PS2) hold simultaneously;
(PS4) (*h*_0_, *h*)-practically asymptotically stable if (PS1) holds and for any *ϵ* > 0 there exists *T*_0_ > 0 such that *t*_0_ + *T*_0_ ∈ *𝕋* and *h*_0_(*t*_0_, *X*_0_) < *λ* implies *h*(*t*, *X*(*t*)) < *ϵ*, *t* ≥ *t*_0_ + *T*_0_.

One can similarly define corresponding notion for the system (22).

**Definition**

Let *λ*, *A*, *B* be positive constants (*λ* < *A*, *B* < *A*), *Q*_0_, *Q* ∈ Σ. Then we say that the system (22) is (*Q*_0_, *Q*)-practically stable if for any 0 < *λ* < *A*, the condition *Q*_0_(||*U*_0_||_*M*_) < *λ* implies *Q*(||*U*(*t*)||_*M*_) < *A*, *t* ≥ *t*_0_, *t* ∈ *𝕋*, where *U*(*t*) = *U*(*t*, *t*_0_, *U*_0_) is any solution of (22).

Other practical stability notions can be defined similarly.

**Theorem 21**

Assume that

(*A*_1_) *λ*, *A* are positive constants and 0 < *λ* < *A*;
(*A*_2_) *h*_0_, *h* ∈ Γ, *t* ∈ *𝕋*, *h*_0_ is nondecreasing in *t*, and *h*_0_ is uniformly finer than *h*, *h*(*t*, *X*) ≤ *ϕ*(*h*_0_(*t*, *X*)), *ϕ* ∈ *𝒦*, where *h*_0_(*t*, *X*) < *λ*;
(*A*_3_) there exists *V* ∈ *C*_*rd*⁡_[*𝕋* × *K*(ℝ^*n*^)^*N*^, ℝ^*N*^], such that *V*(*t*, *X*) is locally Lipschitzian in *X* for each right-dense *t* ∈ *𝕋* and for *a*, *b* ∈ *𝒦*. It holds that
  (31) $$\begin{array}{c} b\left(h\left(t,X\right)\right)\leq {||V\left(t,X\right)||}_{M}\leq a\left(h_{0}\left(t,X\right)\right); \end{array}$$
(*A*_4_) for (*t*, *X*) ∈ *S*(*h*, *A*),
  (32) $$\begin{array}{c} D_{\Delta }^{+}V\left(t,X\right)\leq G\left(t,V\left(t,X\right)\right), \end{array}$$

where *G* ∈ *C*_*rd*⁡_[*𝕋* × ℝ^*N*^, ℝ^*N*^] is upper quasi-monotone nondecreasing in *U* for each *t* ∈ *𝕋* and *U*(*t*) is the solution of (22);

 (*A*_5_) *ϕ*(*λ*) < *A*, *a*(*λ*) < *b*(*A*).

Then practical stability properties of system (22) imply the corresponding (*h*_0_, *h*)-practical stability properties of (18).

**Proof**

Assume that (22) is practically stable; then for given (*a*(*λ*), *b*(*A*)), we have

(33) $$\begin{array}{c} {||U_{0}||}_{M}<a\left(\lambda \right)\text{implying}\;{||U\left(t\right)||}_{M}<b\left(A\right),\;t\geq t_{0}. \end{array}$$

Then by (*A*_2_) and (*A*_5_), it follows that *h*(*t*_0_, *X*_0_) ≤ *ϕ*(*h*_0_(*t*_0_, *X*_0_)) < *ϕ*(*λ*) < *A*. We claim that *h*(*t*, *X*) < *A*.

Indeed, if this were not true, there would exist a solution *X*(*t*) of (18) with *h*_0_(*t*_0_, *X*_0_) < *λ* and *t*_1_ > *t*_0_, *t*_1_ ∈ *𝕋*, such that *h*(*t*_1_, *X*(*t*_1_)) ≥ *A*, *h*(*t*, *X*(*t*)) < *A*, *t*_0_ ≤ *t* < *t*_1_. As *h*_0_(*t*_0_, *X*_0_) < *λ*, Theorem 16 together with (*A*_2_), (*A*_3_) implies that

(34) ||V(t,X)||M<||U(t)||M, t0≤t<t1,||V(t0,X0)||M≤a(h0(t0,X0))<a(λ),b(A)≤b(h(t1,X(t1,X(t1))))  ≤||V(t1,X1)||M≤||U(t1)||M≤b(A).

This contradiction proves that *h*_0_(*t*_0_, *X*_0_) < *λ* implies *h*(*t*, *X*) < *A*, *t* ≥ *t*_0_.

Next we prove that system (18) is (*h*_0_, *h*)-strongly practically stable. For given positive numbers *λ*, *A*, *B*, and *T*, suppose that (22) is strongly practically stable for positive numbers *a*(*λ*), *b*(*A*), *b*(*B*), and *T*; this means we only need to prove (*h*_0_, *h*)-practical quasi-stability of system (18). Practical quasi-stability of (22) means that ||*U*_0_||_*M*_ < *a*(*λ*) implies ||*U*(*t*)||_*M*_ < *b*(*B*), *t* ≥ *t*_0_ + *T* with *t*_0_ + *T* ∈ *𝕋*.

From the foregoing argument, since ||*V*(*t*,*X*(*t*))||_*M*_ < *a*(*λ*), if *h*_0_(*t*_0_, *X*_0_) < *λ*, we have *b*(*h*(*t*, *U*)) ≤ ||*V*(*t*,*X*)||_*M*_ ≤ ||*U*(*t*)||_*M*_ < *b*(*B*) for all *t* ≥ *t*_0_ + *T* if *h*_0_(*t*_0_, *X*_0_) < *λ*; thus we have *h*(*t*, *X*(*t*)) < *B*, *t* ≥ *t*_0_ + *T* provided *h*_0_(*t*_0_, *X*_0_) < *λ*. Hence system (18) is (*h*_0_, *h*)-strongly practically stable.

Finally, we show that system (18) is (*h*_0_, *h*)-practically asymptotically stable. Now, let us suppose that (22) is practically asymptotically stable. This implies we only need to prove that for any given *ϵ* > 0, there exists *T*_0_ > 0 with *t*_0_ + *T*_0_ ∈ *𝕋* such that *t*_0_ + *T*_0_ ≥ *T* and *h*_0_(*t*_0_, *X*_0_) < *λ* implies *h*(*t*, *X*(*t*)) < *B*, *t* ≥ *t*_0_ + *T*_0_ for system (18). Practical asymptotic stability of (22) means that

(35) ||U0||M<a(λ)implies  ||U(t,t0,U0)||M<b(B),t≥t0+T0.

From the argument above, since ||*V*(*t*_0_,*X*_0_)||_*M*_ < *a*(*λ*) whenever *h*_0_(*t*_0_, *X*_0_) < *λ*, we obtain

(36) $$\begin{array}{c} b\left(h\left(t,X\right)\right)\leq {||V\left(t,X\right)||}_{M}\leq {||U\left(t,U\right)||}_{M}<b\left(B\right) \end{array}$$

for all *t* ≥ *t*_0_ + *T*_0_, if *h*_0_(*t*_0_, *X*_0_) < *λ*. Thus we have *h*(*t*, *X*) < *B*, *t* ≥ *t*_0_ + *T*_0_, provided *h*_0_(*t*_0_, *X*_0_) < *λ*. Hence system (18) is (*h*_0_, *h*)-strongly practically stable.

The proof is complete.

**Theorem 22**

Suppose that the conditions of Theorem 21 are satisfied except that condition (A_3_) is replaced by

(*A*_6_) *Q*_0_, *Q* ∈ Σ and for *a*, *b* ∈ *𝒦*
  (37) $$\begin{array}{c} Q\left({||V\left(t,X\right)||}_{M}\right)\geq b\left(h\left(t,X\right)\right),\\ Q_{0}\left({||V\left(t,X\right)||}_{M}\right)\leq a\left(h_{0}\left(t,X\right)\right). \end{array}$$

Then (*Q*_0_, *Q*)-practical stability properties of system (22) imply the corresponding (*h*_0_, *h*)-practical stability properties of the system (18).

**Proof**

Assume that (22) is (*Q*_0_, *Q*)-practically stable. Then we have (*a*(*λ*), *b*(*A*)), such that

(38) Q0(||U0||M)<a(λ)  implies  Q(||U(t,t0,U0)||M)<b(A),t≥t0.

Suppose that the (*h*_0_, *h*)-practical stability of (18) does not hold; then, arguing as in Theorem 16, by (*A*_2_), (*A*_6_), we can show that

(39) Q0(||V(t,X(t,t0,X0))||M)  ≤a(h0(t,X(t,t0,X0)))  ≤a(h0(t1,X(t1,t0,X0)))≤a(λ).

Then using (*A*_6_), we have

(40) b(A)≤b(h(t1,X(t1,t0,X0)))≤Q(||V(t1,X(t1,t0,X0))||M)≤Q(||U(t1,U(t1,t0,U0))||M)<b(A),

which is a contradiction. The proof is complete.
